# Functional imaging with diffusion-weighted MRI for lung biopsy planning: initial experience

**DOI:** 10.1186/1477-7819-12-203

**Published:** 2014-07-10

**Authors:** Marcos D Guimaraes, Edson Marchiori, Bruno C Odisio, Bruno Hochhegger, Almir G V Bitencourt, Charles E Zurstrassen, Chiang C Tyng, Jefferson L Gross, Rubens Chojniak, Myrna C B Godoy

**Affiliations:** 1 Department of Imaging, AC Camargo Cancer Center, Rua Paulo Orozimbo, 726, Aclimação, Zip 01535-001, São Paulo, SP, Brazil; 2 Department of Radiology, Universidade Federal do Rio de Janeiro, Rua Thomaz Cameron, 438,Valparaiso, Zip 25685-120 Petrópolis, RJ, Brazil; 3 Department of Diagnostic Radiology, MD Anderson Cancer Center, 1515 Holcombe Blvd, Zip 77030 Houston, TX, USA; 4 Department of Radiology, Universidade Federal de Ciências da Saúde de Porto Alegre, Rua Prof. Annes Dias, 295, Centro, Zip 90020-090 Porto Alegre, RS, Brazil; 5 Department of Interventional Radiology, AC Camargo Cancer Center, Rua Paulo Orozimbo, 726, Aclimação, Zip 01535-001 São Paulo, SP, Brazil; 6 Department of Thoracic Surgery, AC Camargo Cancer Center, Rua Paulo Orozimbo, 726, Aclimação, Zip 01535-001 São Paulo, SP, Brazil

**Keywords:** Functional imaging, MRI-guided biopsy, DWI, Transthoracic biopsy, Lung lesion, PET/CT-guided biopsy

## Abstract

**Background:**

The purpose of this study was to evaluate the usefulness of diffusion-weighted magnetic resonance imaging (DW-MRI) and positron emission tomography/computed tomography (PET/CT) in planning transthoracic CT-guided biopsies of lung lesions.

**Methods:**

Thirteen patients with lung lesions suspicious for malignancy underwent CT-guided biopsy. Chest DW-MRI and apparent diffusion coefficient (ADC) calculation were performed to aid biopsy planning with fused images. MRI was indicated due to large heterogeneous masses, association with lung atelectasis/consolidation/necrosis, and/or divergent results of other biopsy type and histopathology versus clinical/radiological suspicion. Eight patients underwent PET/CT to identify appropriate areas for biopsy.

**Results:**

Mean patient (*n* = 9 males) age was 59 (range, 30 to 78) years. Based on DW-MRI results, biopsies targeted the most suspicious areas within lesions. All biopsied areas showed higher DW signal intensity and lower ADCs (mean, 0.79 (range, 0.54 to 1.2) × 10^−3^ mm^2^/s), suggesting high cellularity. In patients who underwent PET/CT, areas with higher 18-fluorodeoxyglucose concentrations (standard uptake value mean, 7.7 (range, 3.6 to 13.7)) corresponded to areas of higher DW signal intensity and lower ADCs. All biopsies yielded adequate material for histopathological diagnosis.

**Conclusions:**

Functional imaging is useful for lung biopsy planning. DW-MRI and PET/CT increase overall performance and enable the collection of adequate material for specific diagnosis.

## Background

Percutaneous needle biopsy is considered to be reliable for the evaluation of indeterminate lung lesions. Fine- or cutting-needle biopsies can be performed with computed tomographic (CT) guidance [1-3]. The high sensitivity and specificity of the method and the low rate of complications have established cutting-needle biopsy as an efficient and safe tool for the diagnosis of lung lesions [4]. Despite the high accuracy of this biopsy method, false-negative results can occur, especially in large heterogeneous lesions with necrosis and those with associated postobstructive atelectasis or consolidation.

In the current era of personalized medicine, advances in oncological treatment have been based on targeted therapy and molecular profiling, enabling the collection of tissue specimens from regions representing a lesion’s most aggressive behavior. For this reason, functional/metabolic methods have been sought to enable the identification of the most suspicious areas within a lesion for biopsy. In this context, contrast-enhanced CT and 18-fluorodeoxyglucose (^18^ F-FDG) positron emission tomography (PET)/CT have been used for pre-biopsy lesion evaluation [5].

Recent advances in magnetic resonance imaging (MRI), including the development of new functional methods such as diffusion-weighted imaging (DWI), have expanded the use of this modality for the evaluation of lung lesions [6,7]. DWI evaluates the degree of cellularity throughout a lesion, enabling differentiation of regions with high and low degrees of cellularity through the analysis of water molecule motion [8]. This information can be important for cancer treatment planning and response evaluation [9,10]. MRI can also currently be used for lung cancer screening and staging of lung malignancies [11,12].

The purpose of this study was to evaluate the usefulness of DW-MRI and PET/CT in the planning of transthoracic CT-guided biopsies of lung lesions that are suspicious for malignancy.

## Methods

In this prospective study, 13 patients with pulmonary lung lesions that were suspicious for malignancy based on clinical and imaging findings and who were referred for initial transthoracic CT-guided biopsy at our institution between July 2011 and August 2012, were evaluated. All patients required further MRI evaluation, as determined by the following criteria: 1) presence of multiple or large heterogeneous masses, 2) association of lesions with lung atelectasis or consolidation in the adjacent lung parenchyma, 3) invasion of the chest wall or mediastinal structures, 4) large-vessel involvement, and/or 5) results of biopsy by another method that diverged from clinical and radiological suspicion. No patient had previously undergone CT-guided biopsy that yielded inconclusive results. The time interval of initial CT to pre-biopsy MRI did not exceed 30 days in all cases. Screening coagulation tests were ordered routinely and all patients underwent chest MRI 1 h prior to biopsy to assist in procedure planning. The institutional review board of AC Camargo Cancer Center approved the study and all patients provided written informed consent.

MRI was performed with patients in the supine position using a 1.5-T unit (Signa HDxt; General Electric Medical Systems, Milwaukee, WI, USA) with a body phased-array coil. Before DWI, axial T1- and T2-weighted fast spin-echo images (repetition time/echo time, 6.3/2.7 ms and 3200/40 ms, respectively) with fat signal suppression, 6-mm slice thickness, a 30-cm field of view, and a 256 acquisition matrix were obtained. DWI was performed using a multislice, single-shot, spin-echo and echo-planar imaging sequence in the transverse plane. DW images were acquired with no gated sequence on the basis of prior T1- and T2-weighted images; this sequence was performed only in sections containing the pulmonary lesion during breath holding. Pairs of motion-probing gradients were applied before and after the 180° radiofrequency pulse of the spin-echo T2-weighted sequence in three orthogonal directions (X, Y, and Z). DW images were obtained at diffusion factor (b) values of 0 and 600 s/mm^2^.

Apparent diffusion coefficient (ADC) maps were automatically reconstructed for all DWI, and ADCs were measured after defining regions of interest (ROIs). Regions within the lesions with the lowest ADCs were identified for CT-guided biopsy.

Post-contrast T1-weighted imaging was performed using the same (pre-contrast) parameters and gadopentetate dimeglumine (Magnevist; Berlex Laboratories, Wayne, NJ, USA) paramagnetic contrast. A 20-ml dose was administered at a rate of 2 ml/s using a power injector. For each patient, panoramic acquisition of a scout image was performed with 5 to 10-mm cuts for biopsy planning purposes. Then, DW and CT images were fused using Aquarius NET software (TeraRecon, Foster City, CA, USA) to obtain corresponding plans. All image fusion procedures were very accurate displayed side-by-side, with millimetric adjustments made by overlapping images in the same plane and cut, considering at least three points of common anatomical reference. An approach enabling CT-guided direction of the needle tip to the region of greatest cellularity, as identified by MRI performed 1 h prior to the procedure, was chosen.

Eight patients in this study underwent PET/CT in the week prior to the procedure according to clinical indication. PET/CT studies were interpreted by qualitatively (visually) and quantitatively analyzing areas of anomalous ^18^ F-FDG concentrations; quantitative analysis was performed by measuring standard ^18^ F-FDG values or standardized uptake values (SUVs) in anomalous areas. According to the imaging department’s protocol, PET/CT body scans (Gemini; Philips Medical Systems, Milpitas, CA, USA) were acquired after patients received standard intravenous doses of 560 MBq ^18^ F-FDG. The images were obtained 1 h after radiotracer injection using a high-sensitivity bi-dimensional mode with 10-mm axial cuts and a 256 × 256 matrix. Tomographic image acquisition parameters were 140 kVp, 120 to 200 mA, 0.8 s per CT rotation, with 25-mm cuts and 3-mm thickness. PET and CT images were analyzed together to identify functional and anatomical correlations and sent for reconstruction using specific algorithms. The maximum SUV was calculated by selecting the most representative volumetric ROI within each tumor.

All biopsies were performed with a helical CT unit (HiSpeed; General Electric Medical Systems) using the cutting-needle biopsy technique with an automated 20-gauge coaxial system (Angiotech, Vancouver, Canada). Needles were 10 cm or 15 cm in length, depending on the distance between the skin and the lesion. Local anesthesia was administered routinely, and sedation was not necessary in any case. At least five specimens were collected from the single needle location chosen under MRI (DWI and ADC) and PET/CT (^18^ F-FDG) guidance, when available, in each lesion. Biopsy specimens were subjected to histological evaluation for specific diagnosis.

## Results

Nine of the 13 patients were male and four were female (mean age, 59 (range, 30 to 78) years). Table 1 summarizes patients’ demographics and radiological and histological characteristics of the lesions.

**Table 1:** Demographic data and imaging and histopathological results in patients with lung lesions submitted to percutaneous biopsy based on diffusion-weighted magnetic resonance imaging (DW-MRI) findings

**Age (years), sex**	**Lesion size (mm)**	**ADC**^**a**^	**SUV**	**Histopathological result**
38, male	50	0.61	-	Metastasis from synovial sarcoma
52, male	63	0.80	-	Metastasis from hypopharyngeal SCC
30, male	32	0.78	-	Metastasis from melanoma
58, male	52	1.20	7.0	Carcinoid tumor
31, male	83	0.72	-	High-grade sarcoma
62, female	28	0.79	5.7	Lung adenocarcinoma
69, male	39	0.74	-	Lung SCC
76, female	76	0.99	3.6	Lung adenocarcinoma
76, male	20	0.88	10.1	Lung SCC
74, female	75	0.58	13.7	Lung SCC
78, female	24	0.74	5.5	Lung adenocarcinoma
63, male	67	0.54	9.6	Lung adenocarcinoma
62, male	80	0.96	6.6	Lung adenocarcinoma

^a^Obtained at the lesion biopsy site. ADC, apparent diffusion coefficient; SCC, squamous cell carcinoma; SUV, standard uptake value.

All lung biopsies provided adequate material, allowing for specific histopathological diagnoses: lung adenocarcinoma (*n* = 5), squamous cell carcinoma (*n* = 3), carcinoid tumor (*n* = 1), primary sarcoma (*n* = 1) and metastases (*n* = 3; from synovial sarcoma, hypopharynx adenocarcinoma, and melanoma). No major complication was reported after the procedure.

**Figure 1:**
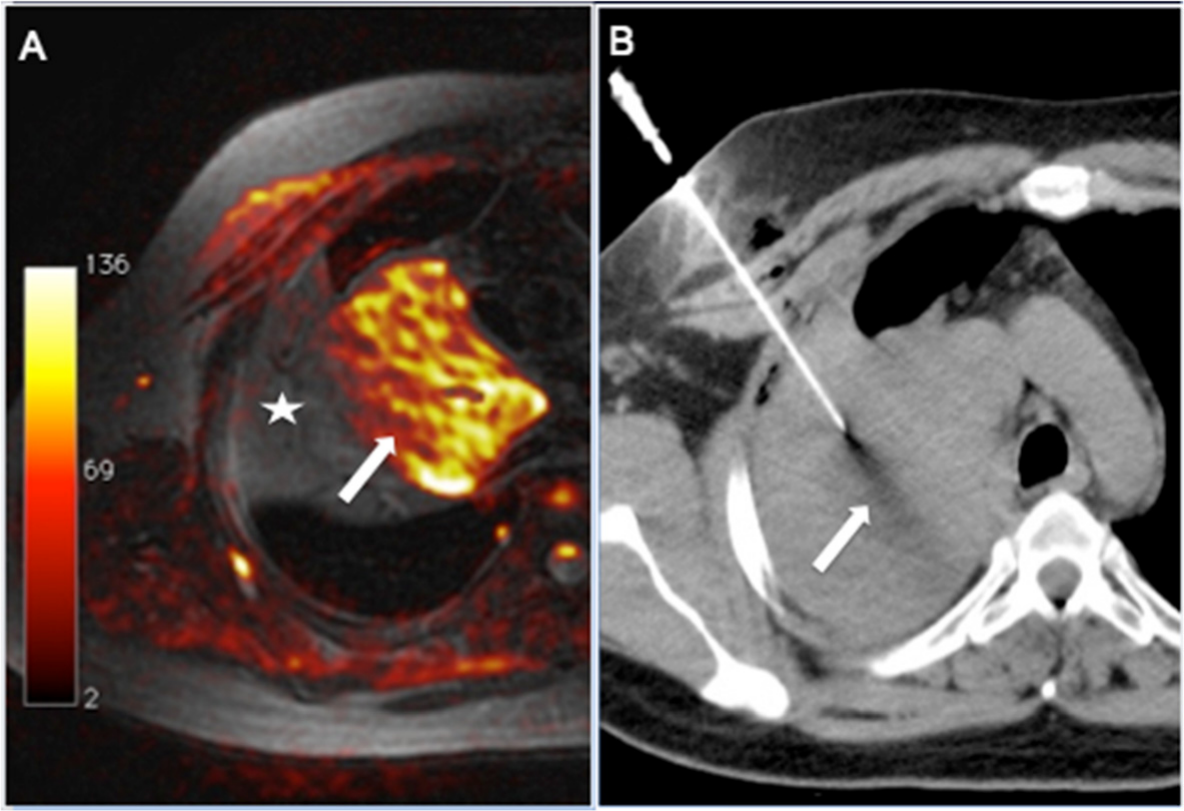
**A 62-year-old man presenting with a heterogeneous mass in the superior lobe of the right lung. (A)**: Fusion of axial fat-saturated T1-weighted and diffusion-weighted sequences. Magnetic resonance imaging (MRI) demonstrated that only the anteromedial portion (arrow) of the mass showed high signal intensity on the diffusion-weighted MRI sequence, suggesting that the rest of the mass may be composed of atelectasis, consolidation, or necrosis. **(B)** Computed tomography (CT)-guided biopsy was directed to this area, and histopathological examination yielded a diagnosis of lung adenocarcinoma.

**Figure 2:**
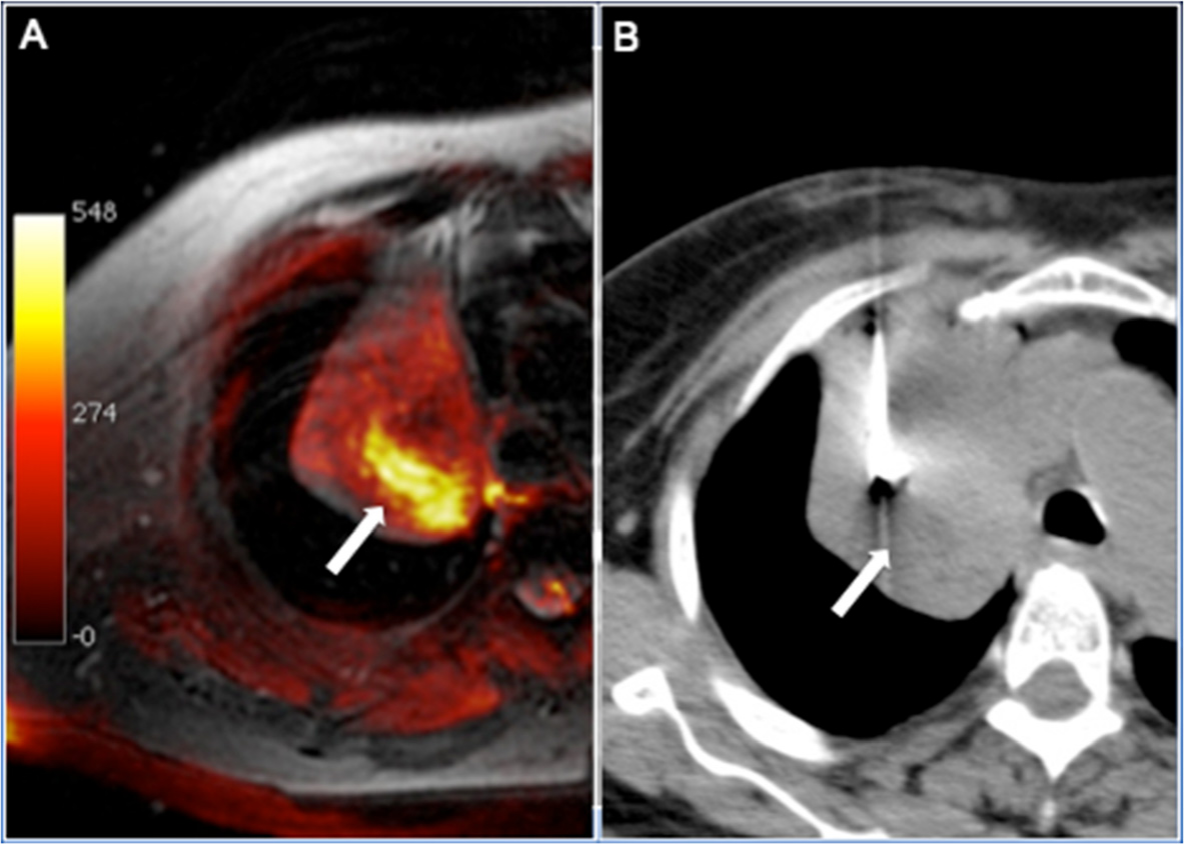
**A 76-year-old woman presenting with a heterogeneous mass in the superior lobe of the right lung. (A)** Fusion of axial fat-saturated T1-weighted and diffusion-weighted sequences. Only the central-posterior portion (arrow) of the mass showed high signal intensity on the diffusion-weighted magnetic resonance imaging (MRI) sequence, suggesting that the rest of the mass may be composed of atelectasis, consolidation, or necrosis. **(B)** Computed tomography (CT)-guided biopsy was directed to this area, and histopathological examination yielded a diagnosis of lung adenocarcinoma.

All biopsied areas showed higher signal intensity on DWI sequences and correspondingly lower ADCs (mean, 0.79 (range, 0.54 to 1.2) × 10^−3^ mm^2^/s) in comparison with other areas within the lesion, suggesting high degrees of cellularity (Figures 1 and 2). Eight patients also underwent PET/CT, and areas of higher ^18^ F-FDG concentrations on PET/CT images (standard uptake value, SUV mean, 7.7 (range, 3.6 to 13.7)) corresponded to areas of higher signal intensity on DWI sequences and lower ADCs in all cases (Table 1).

## Discussion

Within the context of contemporary oncology’s focus on personalized medicine, most treatments are based on targeted therapy and molecular profiling. Interventional radiologists’ ability to collect tissue specimens that are representative of tumor aggressiveness is thus increasingly important. Functional imaging methods, such as DWI and PET/CT, are key to the effectiveness of image-guided percutaneous biopsies, obviating more invasive procedures for proper diagnosis and therapeutic planning [5,13]. In the present study, all biopsies were performed under functional imaging guidance, including PET/CT and DWI, and provided adequate material for analyses. Excellent results were obtained due to the performance of accurate anatomical and functional imaging fusion prior to all procedures to identify areas best representing lesion aggressiveness [14]. This approach enabled CT-guided direction of the needle tip to the region of greatest cellularity, previously identified by MRI and PET/CT.

Biopsy has become a cornerstone of modern medicine, and most modern biopsies are performed percutaneously using image guidance, typically CT or ultrasound [4,15,16]. Recently, the use of MRI has grown to include guidance for minimally invasive and open surgical procedures [16]. The aims of MRI guidance in all of these approaches are to reduce the invasiveness of the procedure and improve the diagnostic yield. MRI has some advantages in relation to other image guidance techniques. First, MRI provides unparalleled soft-tissue contrast and exquisite anatomic detail, enabling the identification of target lesions that cannot be observed with ultrasound or CT and the detailed visualization of anatomy surrounding the target [17]. This level of detail aids in preventing collateral damage to delicate structures that typically would not be well visualized using other imaging modalities. Additionally, MRI provides the unique ability to identify different tissue characteristics during a procedure with the use of different pulse sequences, which can range from simple T1 or T2 weighting to more advanced functional information, such as flow, perfusion, and diffusion [10,18].

In biological tissue, DWI signals are derived from the motion of water molecules in the extracellular, intracellular, and intravascular spaces. The degree of restriction to water diffusion in biologic tissue is inversely correlated with tissue cellularity and cell membrane integrity [6]. Recent studies have evaluated the use of DWI to distinguish malignant from benign lesions [8]. Malignant tumors have high degrees of cellularity with reduced extracellular space, which decreases the diffusion of water, resulting in higher DWI signal intensity and lower ADCs [7,9]. In a review of 34 cases, Tondo *et al*. [8] showed that DWI was useful in differentiating malignant from benign lesions of the lung and mediastinum with 91% diagnostic accuracy, 90% sensitivity, 100% specificity, a 100% positive predictive value, and a 57% negative predictive value. Mori *et al*. [19] compared DWI with PET/CT for the evaluation of lung lesions and found that the methods had similar sensitivities, but DWI had higher specificity and fewer false-positive results than did PET/CT. According to Regier *et al*. [20], ADCs are inversely correlated with maximum SUVs on PET/CT in non-small cell lung cancer. PET/CT can also be used to plan lung lesion biopsies with good diagnostic accuracy [5,21]. Nevertheless, further studies are needed to assess whether DWI has equivalent or better performance than PET/CT in the planning of transthoracic biopsy of lung lesions. As both are considered functional images is possible that there is a strict equivalence; however, this is an assumption. In the present study, all ^18^ F-FDG avidity areas within lesions were correlated with DWI/ADC data in eight patients who underwent PET/CT.

Hoffmann *et al*. [22] reported on the accuracy, duration, and factors influencing the use of MRI-guided biopsies. Liu *et al*. [23] demonstrated that MRI-guided transthoracic biopsy has high levels of accuracy, sensitivity and specificity with 97%, 96% and 100%, respectively, in the diagnosis of small malignant nodules of less than 2.0 cm. However, DWI can be time consuming and costly. Thus, its use should be considered in complex cases, including large, heterogeneous, and mixed-pattern lesions or in severely debilitated patients. The time and cost requirements of this modality are justified in these special cases because the collection of inadequate material for analysis necessitates repetition of the procedure, increasing the risk to the patient and overall cost. Another advantage of MRI is the absence of risks of ionizing radiation, which is usually present in the procedures performed by traditional methods such as CT image and the PET/CT [24].

A few limitations of this study should be emphasized. First, the patient sample was small. Second, no patient underwent control or follow-up chest MRI. Third, the establishment of an ADC cutoff value was not possible. However, to our knowledge, this preliminary study is the first to demonstrate the feasibility of using DW-MRI and ADC in pulmonary lesions for procedure planning. Additional studies are necessary to further evaluate the impact of this technique on the diagnostic yield of transthoracic lung biopsy, particularly in patients with large lesions and/or severe illnesses.

## Conclusions

In conclusion, functional imaging is a useful tool in lung biopsy planning. DW-MRI and PET/CT increase overall performance and enable the collection of adequate material for specific diagnosis.

## Abbreviations

ADC: apparent diffusion coefficient; CT: computed tomography; DWI: diffusion-weighted imaging; MRI: magnetic resonance imaging; PET/CT: positron emission tomography/computed tomography; ROI: region of interest; SCC: squamous cell carcinoma; SUV: standard uptake value.

## Competing interests

The authors declare that they have no competing interests.

## Authors’ contributions

We confirm that each author participated sufficiently in this submission to take public this content. MDG, EM, AB, BO and MCBG contributed to the design of the study, statistics, data interpretation, literature review, and final revision of the manuscript. BH, CZ, CT, JG and RC contributed to the collection of the data, literature review, and preparation of the manuscript. This manuscript publication was approved by all named authors. All authors read and approved the final manuscript.
